# Screening for differentially expressed miRNAs in *Aedes albopictus* (Diptera: Culicidae) exposed to DENV-2 and their effect on replication of DENV-2 in C6/36 cells

**DOI:** 10.1186/s13071-018-3261-2

**Published:** 2019-01-18

**Authors:** Jianxin Su, Gang Wang, Chunxiao Li, Dan Xing, Ting Yan, Xiaojuan Zhu, Qinmei Liu, Qun Wu, Xiaoxia Guo, Tongyan Zhao

**Affiliations:** 1 State Key Laboratory of Pathogen and Biosecurity, Beijing Institute of Microbiology and Epidemiology, Beijing, 100071 People’s Republic of China; 2 Center for Disease Control and Prevention of Guangzhou Military Region, Guangzhou, 510507 People’s Republic of China; 3 Hangzhou Customs District, Hangzhou, 310012 People’s Republic of China

**Keywords:** *Aedes albopictus*, microRNA (miRNA), Midgut, Dengue virus (DENV)

## Abstract

**Background:**

The mosquito *Aedes albopictus* is an important vector for dengue virus (DENV) transmission. The midgut is the first barrier to mosquito infection by DENV, and this barrier is a critical factor affecting the vector competence of the mosquito. However, the molecular mechanism of the interaction between midgut and virus is unknown.

**Results:**

Six small libraries of *Ae. albopictus* midgut RNAs were constructed, three of which from mosquitoes that were infected with DENV-2 after feeding on infected blood, and another three that remained uninfected with DENV-2 after feeding on same batch of infected blood. A total of 46 differentially expressed miRNAs were identified of which 17 significant differentially expressed miRNAs were selected. Compared to microRNA expression profiles of mosquitoes that were uninfected with DENV-2, 15 microRNAs were upregulated and two were downregulated in mosquitoes that were infected with DENV-2. Among these differentially expressed microRNAs, miR-1767, miR-276-3p, miR-4448 and miR-622 were verified by stem-loop qRT-PCR in samples from seven-day-infected and uninfected midguts and chosen for an *in vitro* transient transfection assay. miR-1767 and miR-276-3p enhanced dengue virus replication in C6/36 cells, and miR-4448 reduced dengue virus replication.

**Conclusions:**

To our knowledge, this study is the first to reveal differences in expression levels between mosquitoes infected and uninfected with DENV-2 after feeding on an infected blood meal. It provides useful information on microRNAs expressed in the midgut of *Aedes albopictus* after exposure to the virus.

**Electronic supplementary material:**

The online version of this article (10.1186/s13071-018-3261-2) contains supplementary material, which is available to authorized users.

## Background

The mosquito *Aedes albopictus* is an important vector for transmission of many arboviruses, including dengue virus (DENV) which causes dengue fever (DF), with some cases resulting in severe symptoms such as plasma leakage, hemorrhagic fever and organ impairments. Approximately four billion people in 128 countries are estimated to be at risk of DENV infection [1]. Each year, there are approximately 390 million cases of dengue fever worldwide, many of which are asymptomatic or undiagnosed [2]. Because there is no reliable vaccine for dengue fever and no drug therapies exist, vector control is still the main effective means to prevent this disease. However, for many reasons, for example the emergence of insecticide-resistant mosquitoes, lower impact of prevention and control efforts than in the past [3], and increasing vector and human population densities, the global pandemic of dengue fever has increased dramatically in recent decades [4], emphasizing the need for diversification of vector control strategies. Previous studies have carried out genetic manipulation of insect vectors to modulate characteristics such as vector competence [5, 6], but for further in-depth researches, more knowledge of the molecular mechanism of vector-arbovirus interactions is needed.

The susceptibility of mosquito vectors to the virus is the principal factor for vector competence. A large number of studies have shown that the mosquito innate immune response is activated after mosquitoes are infected by various pathogens [7–10]. Molecular events triggered by the innate immune system either prohibit the infection of virus in the midgut epithelium (e.g. a midgut infection barrier, MIB) or prevent virus escape and dissemination to other tissues, like salivary glands and ovaries (e.g. a midgut escape barrier, MEB) [11]. The midgut is the primary barrier to pathogens invading *via* the digestive tract, so the antiviral ability of midgut epithelial cells is the most important factor affecting the susceptibility of mosquitoes to arboviral infection and the crucial indicator of vector competence [12]. At present, the molecular mechanism by which midgut epithelial cells regulate viral replication remains unclear, which is a major obstacle to studying the susceptibility of mosquitoes to DENV.

MicroRNAs (miRNAs) are a class of non-coding RNAs that regulate gene expression at the post-transcriptional level [13, 14]. miRNA plays an important role in regulating endogenous genes and resisting the invasion of exogenous nucleic acids. In recent years, many studies have shown that miRNA plays an important role in the growth, development and infection of animals [15, 16]. To date, more than 100 miRNAs have been identified within mosquitoes (miRBase20, http://www.mirbase.org/). Several studies have demonstrated that DENV infection causes changes in the expression of miRNAs, such as miR-375, which is only expressed after a blood meal, in the midgut of *Ae. aegypti* [17, 18]. Transfecting miR-375 mimics into Aag2 cells significantly enhanced the expression of the catus gene, the gene affected DENV infection by inhibiting the expression of the transcription factor NF-κB. Upon further infecting Aag2 cells with DENV-2, miR-375 enhanced DENV-2 infection in the Aag2 cell line [19, 20]. In *Culex quinquefasciatus*, miR-92, miR-33, miR-970 and miR-980 were upregulated and miR-989 and miR-957 were downregulated after West Nile virus (WNV) infection [21]. These results suggest that miRNA may be involved in the process of endogenous gene regulation during pathogen infection, and the process may differ in different combinations of mosquito species and viruses.

Although some *Ae. albopictus* miRNAs have previously been identified, there are few studies comparing the differential expression of miRNAs between DENV-2 infected and uninfected midguts [22, 23]. Due to the importance of the midgut in viral infection, diagnosing the patterns and functions of different miRNAs during the course of DENV infection can help identify the molecular mechanisms of the midgut infection barrier, so the prevention and control of dengue fever can be further improved.

Previously, we constructed three small RNA libraries from the midguts of *Ae. albopictus* females that were either fed sugar solution, a regular blood meal, or a blood meal infected with DENV-2. We then investigated the differences between mosquitoes that had ingested a uninfected blood meal and those that had ingested a DENV-2-infected blood meal [24]. Here, we present the changes in miRNA expression levels between *Ae. albopictus* uninfected and infected with DENV-2, which both feeding on the same batch of infected blood meal. We identified the miRNAs that play an important role in DENV-2 infection. Then, we chose four miRNAs for an *in vitro* transient transfection assay to understand the effect of miRNA on the replication of DENV-2 in the C6/36 cell line.

## Results

Six groups of data were constructed: 5-day infected midguts after exposure to DENV-2 (5A), 5-day uninfected midguts after exposure to DENV-2 (5B), 7-day infected midguts (7A), 7-day uninfected midguts (7B), 10-day infected midguts (10A) and 10-day uninfected midguts (10B).

### Bioinformatic analysis of samples

The results of the analysis of total RNA concentration and integrity showed that the samples meet the requirements for sample preparation (Table 1). The number of sRNAs and the proportion of each sequence are shown in Additional file 1: Table S1. We aligned clean sequences with sequences for rRNA, tRNA, snRNA, snoRNA, repeats, exons and introns in the NCBI GenBank and miRbase and annotated each sequence (see Additional file 2: Table S2). Comparison of the clean sequences with the published *Ae. aegypti* genome (www.vectorbase.org) revealed that approximately 4.84–7.58% of the assembled RNA and approximately 11.76–31.79% of the RNA reads matched that of *Ae. aegypti* (see Additional file 3: Table S3)*.*

**Table 1:** Concentration and quality of total RNAs extracted from the midguts of DENV-2-infected and uninfected *Ae. albopictus*

Sample	Concentration (ng/μl)	Volume (μl)	Total (μg)	RIN	28S:18S
5A	597.0	25	14.925	6.0	0
5B	975.0	25	24.375	5.7	0
7A	525.0	25	13.125	6.6	0
7B	942.0	25	23.55	6.1	0
10A	660.0	25	16.5	6.1	0
10B	1254.0	25	31.35	6.6	0

### Conserved and novel miRNAs

No change was observed in the conserved miRNA compared with the results of our previous study by Su et al. [24]. Meanwhile, we predicted 2 novel miRNAs, aal-miR-10A-m0027-5p (AATTTTGACACTAGAGCGGGG) and aal-miR-10A-m0028-5p (AACGGTCTAGGGTTCATGTC).

### Changes in miRNA expression profiles following DENV infected and uninfected

**Fig. 1:**
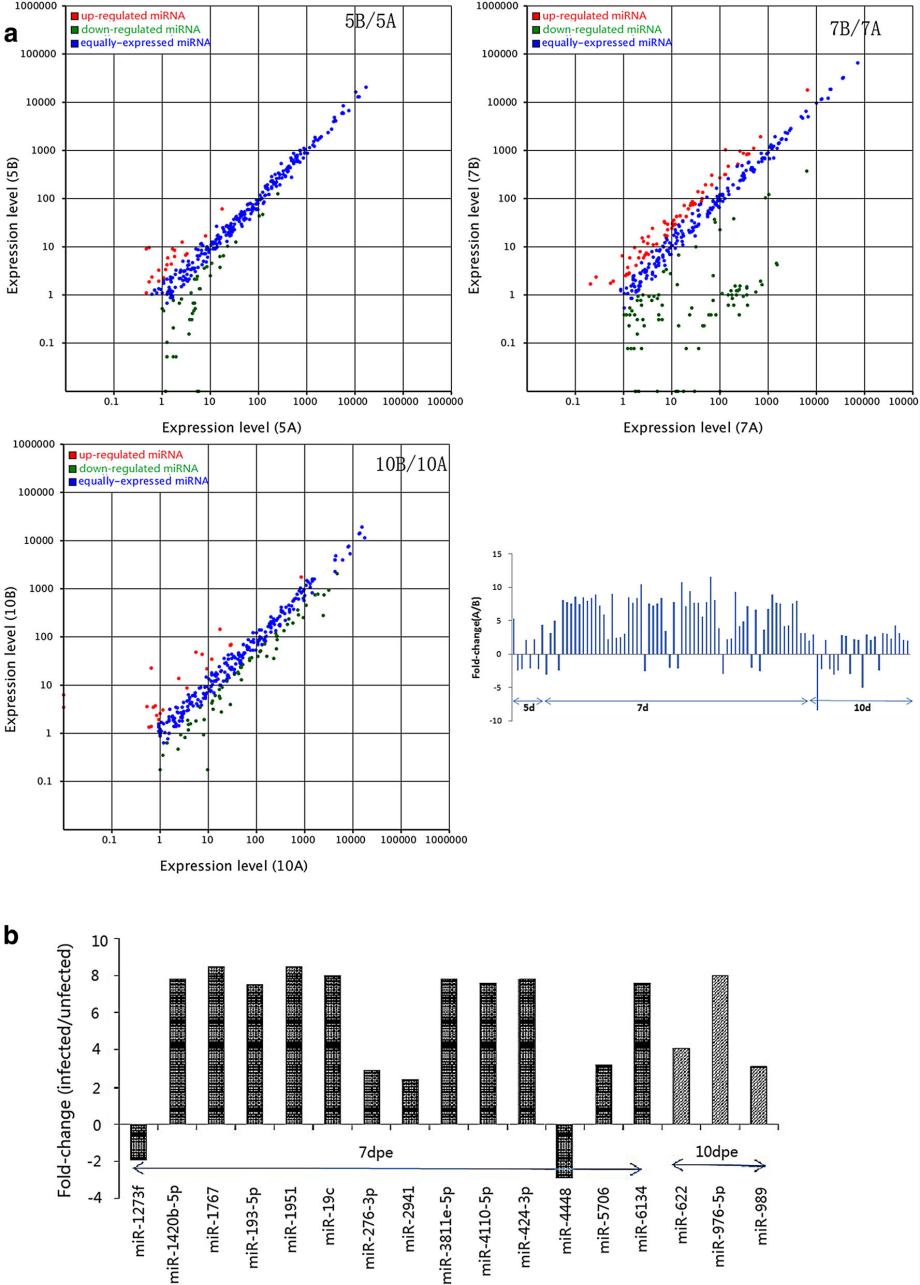
**a** Scatter plots and fold changes comparing the midguts of infected and uninfected *Ae. albopictus* at different time points. 5B/5A is the (5-day uninfected midguts)/(5-day infected midguts after exposure to DENV-2), 7B/7A is the (7-day uninfected midguts)/(7-day infected midguts), etc. **b** Screened significantly differentially expressed miRNAs between the midguts of infected and uninfected *Ae. albopictus* at different time points after a DENV-2-infected blood meal

We normalized the expression of each miRNA (see Additional file 4: Table S4) and calculated the infected and uninfected mosquito midgut miRNA expression ratio. We further analyzed the expression data by calculating the fold changes and *P*-values based on the expression ratios and plotted these data as a scatter plot. Changes after exposure to DENV-2 were apparent, and the differences at 7 days were the most significant (Fig. 1a). The upregulated miRNAs were more numerous than the downregulated miRNAs.

A total of 46 differentially expressed miRNAs were identified (see Additional file 5: Table S5). Using a fold change > 2 and a normalized expression > 30 to screen the miRNAs, 17 miRNAs were selected from the microRNA expression profiles of infected and uninfected midguts. Of these miRNAs, 15 were upregulated (miR-1420b-5p, miR-1767, miR-193-5p, miR-1951, miR-19c, miR-276-3p, miR-2941, miR-3811e-5p, miR-4110-5p, miR-424-3p, miR-5706, miR-6134, miR-622, miR-976-5p and miR-989), and 2 were downregulated (miR-1273f and miR-4448). Fourteen significantly differentially expressed miRNAs appeared 7 days after the DENV-2 blood meal (Fig. 1b).

### Effect of miRNAs on replication of DENV-2 in the C6/36 cell line

**Fig. 2:**
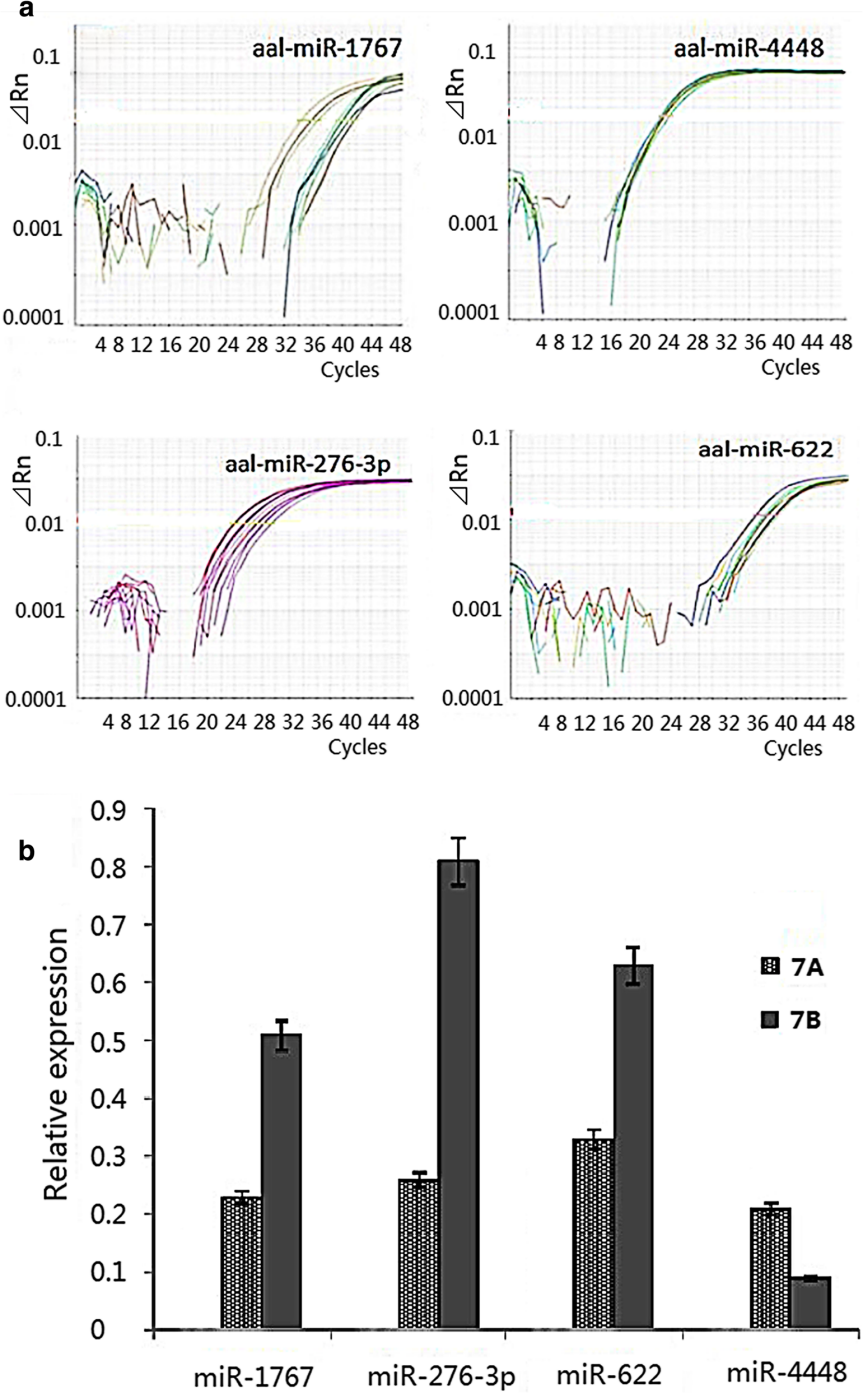
Verification of differentially expressed miRNAs by stem loop qRT-PCR. **a** Amplification curves from stem loop qRT-PCR. **b** Relative expression of miRNA by stem loop qRT-PCR

In this study, four miRNAs (aal-miR-1767, aal-miR-276-3p, aal-miR-4448 and aal-miR-622) were selected for their high expression levels, high degree of fold change between the uninfected and infected midguts, and the repeatability of their differential expression at different time points for the *in vitro* transient transfection assay. The differential expression of these four miRNAs in the samples from the 7-day infected and uninfected midguts were verified by stem-loop quantitative real-time PCR (qRT-PCR) (Fig. 2).

**Fig. 3:**
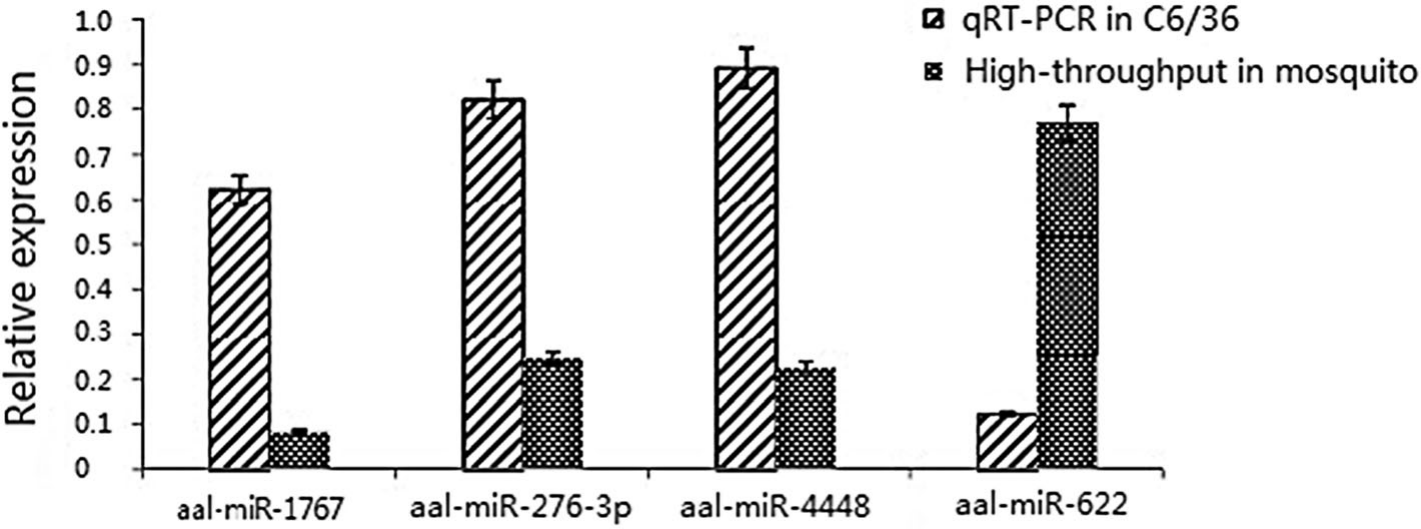
Relative expression of the four miRNAs in C6/36 by stem loop qRT-PCR and in the midguts of *Ae. albopictus* by high throughput sequencing

We transfected C6/36 cells with synthetic mimics of the selected miRNAs and their inhibitors as well as negative controls for the mimic (NCm) and inhibitor (NCi). The cells were inoculated with DENV-2 24 h post-transfection. The transfection efficiency of the negative controls for the mimic (NCm) after 0 h, 6 h, 1 day, 3 days, 5 days and 7 days was 0.796, 14.4, 73, 57.7, 52.3 and 38.8%, respectively. The *Ae. albopictus* housekeeping gene rpS7 was used as an internal control for qRT-PCR, and the four miRNAs were expressed in the *Ae. albopictus* C6/36 cell line (Fig. 3).

**Fig. 4:**
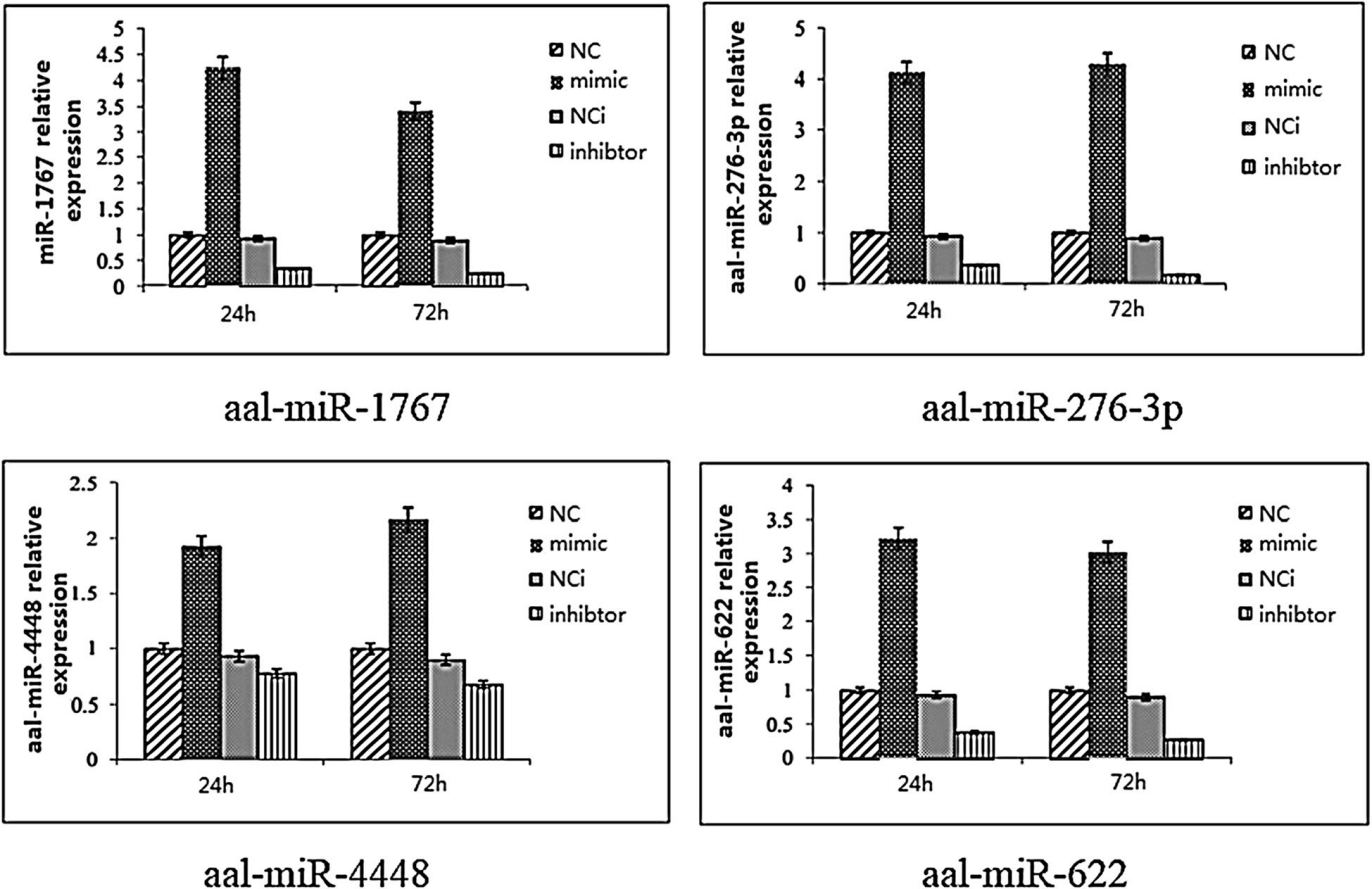
Relative expression of miRNAs in C6/36 cells at 24 and 72 h post-transfection by qRT-PCR

Expression levels of the miRNAs were measured 24 and 72 h post-transfection, and the expression level of DENV-2 E protein was measured using DENV-2 specific primers 72 h post-inoculation. The qRT-PCR results indicated that at 24 and 72 h post-transfection with their mimic, the expression of aal-miR-1767 increased by 4.25-fold *(t*_(2)_ = 9.200, *P* = 0.012) and 3.41-fold (*t*_(2)_ = 134.871, *P *< 0.0001), aal-miR-276-3p increased by 4.13-fold (*t*_(2)_ = 18.210, *P* = 0.003) and 4.29-fold (*t*_(2)_ = 28.633, *P* = 0.001), aal-miR-4448 increased by 1.92-fold (*t*_(2)_ = 29.160, *P* = 0.001) and 2.17-fold (*t*_(2)_ = 63.451, *P* = 0.073), and aal-miR-622 increased by 3.23-fold (*t*_(2)_ = 26.386, *P* = 0.001) and 3.03-fold (*t*_(2)_ = 25.663, *P* = 0.002), respectively. The differences were statistically significant. At 24 and 72 h post-transfection with their inhibitor, the expression of aal-miR-1767 decreased by 0.37-fold (*t*_(2)_ = 36.833, *P* = 0.001) and 0.28-fold (*t*_(2)_ = 29.160, *P* = 0.001), aal-miR-276-3p decreased 0.39-fold (*t*_(2)_ = 16.958, *P* = 0.003) and 0.19-fold (*t*_(2)_ = 17.961, *P* = 0.003), and aal-miR-622 decreased 0.41-fold (*t*_(2)_ = 11.668, *P* = 0.007) and 0.31-fold (*t*_(2)_ = 136.583, *P *< 0.0001), respectively. The differences were statistically significant. The expression of aal-miR-4448 decreased by 0.78-fold (*t*_(2)_ = 0.229, *P* = 0.089) and 0.68-fold (*t*_(2)_ = 3.548, *P* = 0.073), but the difference was not statistically significant (Fig. 4).

Relative expression of DENV-2 was significantly increased in the aal-miR-1767 mimic-transfected group (2.43 times; *t*_(2)_ = 8.325, *P* = 0.014) and the aal-miR-276-3p mimic-transfected group (1.75 times; *t*_(2)_ = 4.887, *P* = 0.039) compared with the NCm group. Transfection with the inhibitors to those two miRNA mimics had the opposite effect on the relative expression of DENV-2, significantly decreasing it in the aal-miR-1767 inhibitor-transfected group by 0.22-fold (*t*_(2)_ = -7.237, *P* = 0.019) and in the aal-miR-276-3p inhibitor-transfected group by 0.49-fold (*t*_(2)_ = -6.434, *P* = 0.023) compared with the Nci group.

**Fig. 5:**
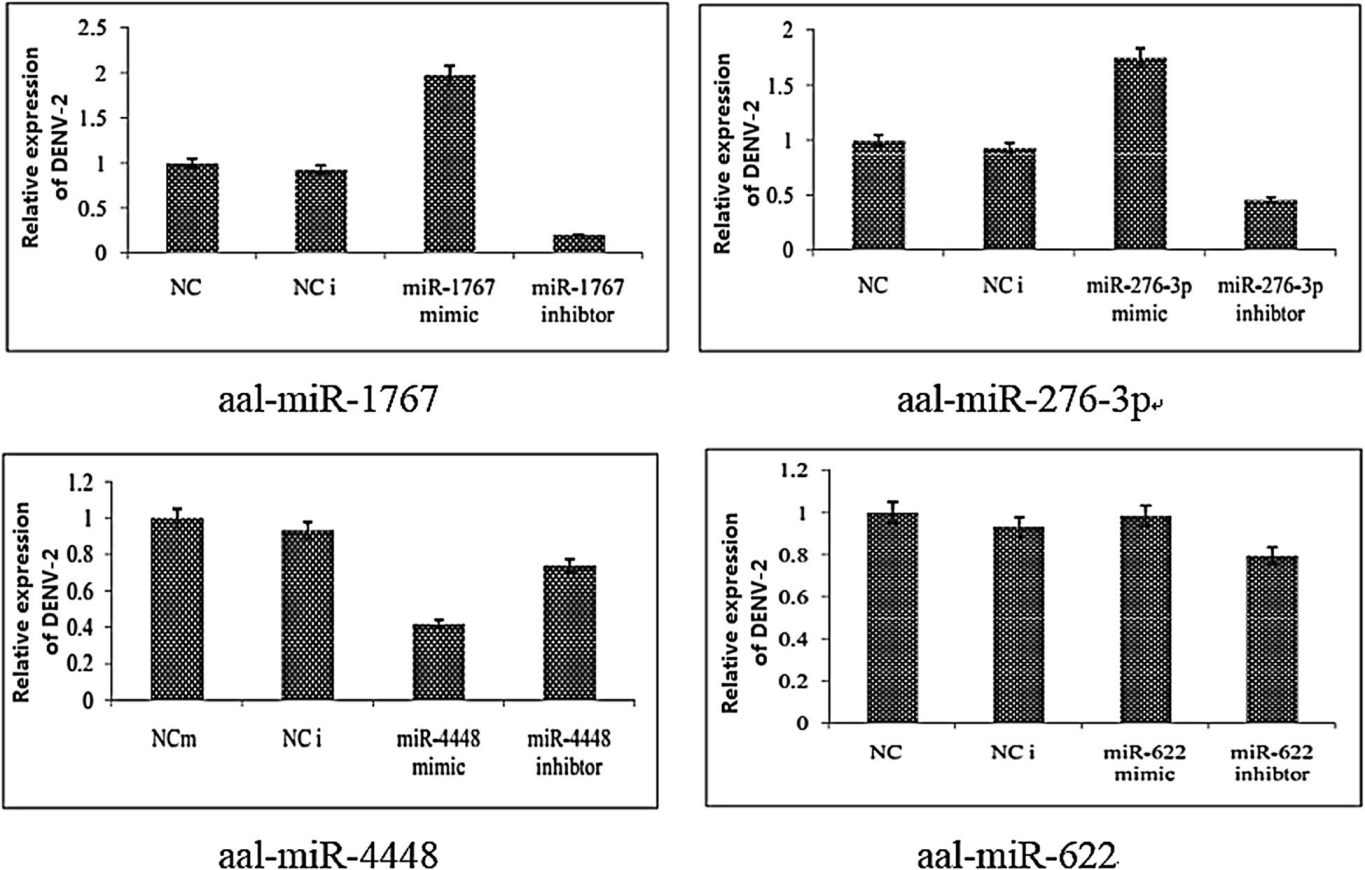
Relative expression of DENV-2 in C6/36 cells at 72 h post-transfection by qRT-PCR

Relative expression of DENV-2 decreased in the aal-miR-4448 mimic-transfected group by 0.42-fold (*t*_(2)_ = -23.432, *P* = 0.012), and the difference was statistically significant. The results suggested that aal-miR-4448 can inhibit DENV-2 replication in C6/36 cells. Relative expression of DENV-2 did not significantly differ between the aal-miR-622 mimic- and inhibitor-transfected cells (0.98 and 0.622, respectively) (Fig. 5).

Titers of DENV-2 in the supernatant of the aal-miR-1767 mimic and aal-miR-276-3p mimic groups were higher (*t*_(2)_ = 6.645, *P* = 0.022; *t*_(2)_ = 7.015, *P* = 0.020) than those of the NCm group, while the DENV-2 titer in the supernatant of the aal-miR-4448 group was lower (*t*_(2)_ = 63.245, *P* = 0.001) (Table 2).

**Table 2:** DENV-2 titers in C6/36 cells at 72 h post-transfection by the TCID50 method

miR name	Item	DENV	NCm	Mimic
miR-1767	TCID50	10^-2.0^/0.1 ml	10^-1.875^/0.1 ml	10^-3.25^/0.1 ml
	TCID50	10^-1.675^/0.1 ml	10^-1.675^/0.1 ml	10^-3.375^/0.1 ml
	TCID50	10^-1.75^/0.1 ml	10^-1.675^/0.1 ml	10^-3.375^/0.1 ml
	Mean ± SD	0.0163059 ± 0.00571247	0.0185350 ± 0.00450315	0.0005311 ± 0.00010940
	*t*-value	–		6.645
	*P-*value	–		0.022
miR-276-3p	TCID50	10^-2.0^/0.1 ml	10^-1.875^/0.1 ml	10^-3.5^/0.1 ml
	TCID50	10^-1.675^/0.1 ml	10^-1.675^/0.1 ml	10^-3.375^/0.1 ml
	TCID50	10^-1.75^/0.1 ml	10^-1.675^/0.1 ml	10^-3.625^/0.1 ml
	Mean ± SD	0.0163059 ± 0.00571247	0.0185350 ± 0.00450315	0.00032503 ± 0.00009259
	*t-*value	–		7.015
	*P-*value	–		0.020
miR-4448	TCID50	10^-2.0^/0.1 ml	10^-1.875^/0.1 ml	10^-1.0^/0.1 ml
	TCID50	10^-1.675^/0.1 ml	10^-1.675^/0.1 ml	10^-1.25^/0.1 ml
	TCID50	10^-1.75^/0.1 ml	10^-1.675^/0.1 ml	10^-0.75^/0.1 ml
	Mean ± SD	0.0163059 ± 0.00571247	0.0185350 ± 0.00450315	0.0613522 ± 0.054097
	*t-*value	–		63.254
	*P-*value	–		0.001

**Fig. 6:**
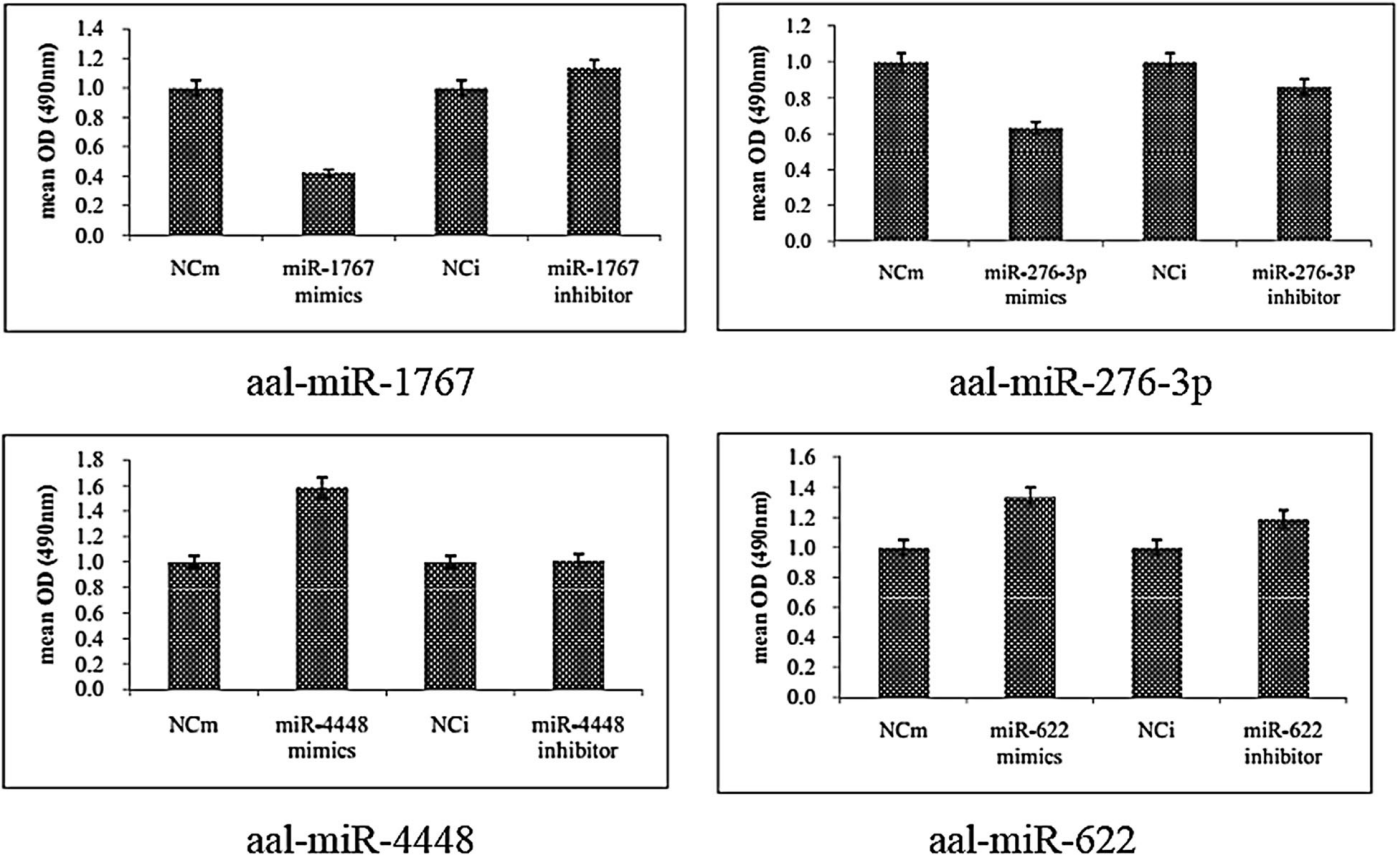
Survival rates of C6/36 cells at 72 h post-infection with DENV-2 by MTT

The survival rate of C6/36 cells was lower in cells transfected with the aal-miR-1767 mimic compared to those transfected with the NCm four days post-inoculation (*t*_(2)_ = 24.137, *P *< 0.0001). The survival rate of C6/36 cells was higher in cells transfected with the aal-miR-1767 inhibitor, but the difference was not statistically significant. The survival rate of C6/36 cells was lower in cells transfected with the aal-miR-276-3p mimic (*t*_(2)_ = 5.679, *P* = 0.011) but was not significantly different in the inhibitor group. The survival rate of C6/36 cells was higher in cells transfected with the aal-miR-4448 mimic (*t*_(2)_ = 24.137, *P *< 0.0001) but was not significantly different in the inhibitor group. The survival rate of C6/36 cells was higher in the aal-miR-622 mimic group but was not significantly different in the inhibitor group (Fig. 6).

## Discussion

The relationship between mosquito and arbovirus is a dynamic process, influenced by the inherent variability of the individual genotypes of both the mosquito and the mosquito-borne virus, and infection is an external expression of those genotype interactions [25–28]. In this study, the difference in miRNA expression between the infected and uninfected *Ae. albopictus* midgut was the most significant at seven days, highlighting the potential importance of this period during infection.

Our results show that many miRNAs are differentially expressed between uninfected and infected midguts at different time points, and the number and amplitude of miRNAs that were upregulated in infected midguts was larger than the number and amplitude of miRNAs that were downregulated. A total of 44 differentially expressed miRNAs were screened. The identification, screening and function of *Ae. albopictus* midgut-specific microRNAs related to susceptibility to dengue virus were observed to be very complex. Some miRNAs showed a trend of upregulation and downregulation at different time points, such as aal-miR-2941 and aal-miR-989.

In *Ae. aegypti* exposed to DENV-2, there were 34 differentially expressed miRNAs that were screened, of which miR-5119-5p was upregulated at two days, miR-34-3p, miR-87-5p and miR-988-5p were upregulated at nine days, and the remaining miRNAs were downregulated [19]. In this study, the expression of miR-34 was upregulated after 24 h of infection, and there was no significant difference compared with uninfected mosquitoes at five days. The expression of miR-87-5p was upregulated after exposure to DENV-2, but the expression level was very low. These results indicate that the expression of the same miRNA varies between different mosquito species. In a study of *Ae. albopictus*, the expression of miR-252 was downregulated at seven days after thoracic injection of DENV-2 [29]. Yan et al. [30] found that the expression of miR-252 was upregulated in C6/36 cells after infection with DENV-2, and the *in vitro* transient transfection assay showed that it inhibited the replication of DENV-2 in C6/36 cells. However, in the present study, the expression of aal-miR-252 in the midgut remained at a very low level and did not change significantly after infection. This implies that aal-miR-252 did not act on the midgut infection barrier. Two factors may be responsible for these different results. One difference between the two studies was the infection mechanism. The present study aimed to simulate the natural infection by using an infected blood meal, and may have triggered a different immune system response compared with thoracic injection. Another factor was that our study focused on the midgut tissue and the whole body. Many studies have shown that there is a difference in the expression pattern of the same miRNA in different tissues [31–33].

Using a fold change of > 2 and a normalized expression of > 30 as screening parameters, 17 miRNAs were identified from microRNA expression profiles of infected and uninfected midguts. Of these miRNAs, 15 were upregulated (miR-1420b-5p, miR-1767, miR-193-5p, miR-1951, miR-19c, miR-276-3p, miR-2941, miR-3811e-5p, miR-4110-5p, miR-424-3p, miR-5706, miR-6134, miR-622, miR-976-5p and miR-989) and two were downregulated (miR-1273f and miR-4448). Fourteen significantly differentially expressed miRNAs appeared at 7 days after the DENV-2 blood meal. The study of the function and effect of these miRNAs on target genes may lead to a breakthrough in elucidating the molecular mechanisms underlying the midgut infection barrier.

In this study, four miRNAs were selected for their high expression levels, high degree of fold change between the uninfected and infected midguts, and the repeatability of their differential expression at different time points for the *in vitro* transient transfection assay. Three of these miRNAs had an effect on DENV-2 replication in C6/36 cells. miR-4448 inhibited intracellular DENV-2 replication, suggesting that it may play an important role in DENV-2 infection in *Ae. albopictus*. aal-miR-1767 and aal-miR-276-3p enhanced the replication of DENV-2. miR-276 was upregulated in *Ae. albopictus* after intraperitoneal injection of DENV-2 [29]. Similarly, in this study, miR-276 was found to be significantly upregulated in the midgut after oral infection, and *in vitro* transient transfection assays demonstrated that it could affect C6/36 intracellular DENV-2 replication, indicating that the miRNA may play an important role in midgut infection with DENV-2. Only one study previously reported that aal-miR-1767 is expressed in the midgut after a sugar meal (109 and 1660 reads) [24]. Our study showed that this miRNA maintained a high level of expression after DENV-2 infection. Furthermore, our *in vitro* transfection experiments demonstrated that miR-1767 enhanced DENV-2 replication in C6/36 cells, indicating that it could be a miRNA that is specifically expressed in the midgut of *Ae. albopictus*. miR-1767 expression was significantly downregulated after chicken fibroblasts were infected with duck virus, indicating that this miRNA could be associated with viral infection [34]. The replication of DENV-2 in C6/36 cells was inhibited after transfection of aal-miR-4448 mimic. A previous study showed that aal-miR-4448 was expressed in the midgut (4459 reads) and was upregulated after an uninfected blood meal but downregulated after the mosquito had ingested a DENV-2 infected blood meal [24]. The results indicated that aal-miR-4448 has an inhibitory effect on DENV-2 infection.

Some studies have also shown that mosquito miRNAs interact with a virus, such as West Nile virus (WNV), which encoded miRNA-like small RNA molecules to upregulate the expression level of GATA4, thereby promoting replication of the virus in mosquito cells [35]. *Wolbachia* can use the miRNAs produced by host cells to regulate the expression of antiviral genes in host cells and play a role in promoting viral replication [36]. These effects may be related to changes in the host cell environment following viral infection and the host’s antiviral mechanisms, but the molecular mechanism is unclear. miR-4448 was also found to be expressed in *Culex pipiens pallens*, and the expression level of sensitive strain was higher than that of resistant strain [37]. The same miRNA may play a different role in different species. miR-4448 may play an important role in DENV-2 infection in *Ae. albopictus*, which is worthy of further study.

The expression of aal-miR-662 was higher in the infected midgut at different time points, but the *in vitro* transient transfection experiment did not show a direct effect on the replication of dengue virus. If not directly on the virus itself, it is likely to have synergistic effects with other genes or may play a role in the midgut escape barrier.

Mosquito genes influence the mosquito’s susceptibility to the virus, and the genotype of the mosquito may affect the choice of control strategies and may eventually provide clues to interrupt transmission [38]. In this study, we first compared the miRNA expression in the *Ae. albopictus* midgut when infected or uninfected with DENV-2. Examination of the miRNA expression profile illustrates the changes of midgut miRNA after infection with DENV-2. We screened the differentially expressed miRNA and chose four miRNAs that may play a role in DENV-2 infection for *in vitro* transfection experiments. Our results provide a basis for further study of novel vector control and measures to block pathogen transmission.

## Conclusions

Six small libraries of *Ae. albopictus* midgut RNAs were constructed, three of which from mosquitoes that were infected with DENV-2 after feeding on infected blood, and another three that remained uninfected with DENV-2 after feeding on the same batch of infected blood. Compared to microRNA expression profiles of mosquitoes that were uninfected with DENV-2, 15 microRNAs were upregulated and two were downregulated in mosquitoes that were infected with DENV-2. Among these differentially expressed microRNAs, miR-1767, miR-622, miR-4448 and miR-276-3p were verified and chosen for an *in vitro* transient transfection assay; miR-1767 and miR-276-3p enhanced dengue virus replication in C6/36 cells, and miR-4448 reduced dengue virus replication.

## Methods

### Mosquito, virus strains, cell and RNA extraction

Methods of mosquito collection and husbandry, mosquito dissection, RNA extraction, small RNA libraries construction, cell culture and cell infection are identical to those used by Su et al. [24]; the same viral strains were also used.

### qRT-PCR

Methods follow Su et al. [24]. After mosquito feeding on a blood meal infected with DENV-2, dengue virus II universal primers were used to detect whether mosquitoes were infected with viruses; the amplification product was 511 bp. The primers were F (5'-TCA ATA TGC TGA AAC GCG CGA GAA ACC G-3') and R (5'-TTG CAC CAA CAG TCA ATG TCT TCA GGT TC-3'). The specific miR-622 stem-loop primer was designed by BGI (Shenzhen, China): 5'-GTC GTA TCC AGT GCG TGT CGT GGA GTC GGC AAT TGC ACT GGA TAC GAC GCC TCC-3'.

### Alignment of conserved miRNAs using BLAST and tag2 miRNA software

To make sure every unique small RNA is mapped to only one annotation, we adhered to the following priority rule: all rRNA (in which GenBank > Rfam) > repeat > exon > intron > known miRNA. Because *Ae. albopictus* miRNA is not available in miRBase, we first used BLAST to align small RNA tags with the *Ae. aegypti* miRNA precursor in miRBase 19.0 to obtain a miRNA count with no mismatches.

### Prediction of novel miRNA candidates using MIREAP software

MIREAP software (http://sourceforge.net/projects/mireap/) was used to predict novel miRNAs by exploring their secondary structure, Dicer cleavage sites and the minimum free energy of unannotated small RNA tags.

### Analysis of variations in miRNA expression

To compare the miRNA expression levels in two groups *via* high throughput deep sequencing, we normalized the read numbers in each library according to the following formula: Normalized expression = Actual miRNA reads/Total count of clean reads × 10^6^. We then calculated the ratio and magnitude of the between-group differences and their associated *P*-values from the normalized data. The ratio was calculated according to the following formula: ratio = normalized expression of treatment group/normalized expression of control group, and the magnitude of the differences was expressed as “fold change” calculated using the following formula: fold change = log_2_ratio. A fold change > 1 or < -1 with a *P*-value < 0.05 was regarded as significantly different [39]. Fold changes and their associated *P*-values were calculated using a special procedure developed by the BGI biotech company (Shenzhen, China).

### rpS7 q-RTPCR

The *Ae. albopictus* housekeeping rpS7 gene was used as an internal control for the qRT-PCR results. The forward primer sequence was 5'-ATG GTT TTC GGA TCA AAG GT-3', and the reverse sequence was 5'-CGA CCT TGT GTT CAA TGG TG-3'. qRT-PCR analyses followed the protocols described above.

### Survival rate of C6/36 cells detection

Twenty microliters of MTT solution (5 mg/ml) was added into a well for 4 h (37 °C, 5% CO_2_), the culture solution was removed, 150 μl of DMSO was added, and the mixture was shaken horizontally for 10 min. The absorbance value at 492 nm was detected by an enzyme labeling instrument (SuPerMax 3000FA).

### Statistical analysis

A Poisson distribution was used to analyze a digital transcript of the profile data following the method described by Audic & Claverie [39]. The 2^-△△CT^ method was used to determine relative expression levels from the qRT-PCR results, and paired t-tests were used to determine if the differences were statistically significant using SPSS v.19.0.

## Additional files

Additional file 1: **Table S1.** Basic biological information analysis on sRNAs in the midguts from infected and uninfected *Ae. albopictus* after a DENV-2-infected blood meal. (DOCX 18 kb)

Additional file 2: **Table S2.** Annotations of clean sRNAs from the midguts of infected *Ae. albopictus* after a DENV-2-infected blood meal. (DOCX 21 kb)

Additional file 3: **Table S3.** Matching of small RNAs on the genome of *Ae. aegypti* in the midguts of infected and uninfected *Ae. albopictus* at different time points after a DENV-2-infected blood meal. (DOCX 15 kb)

Additional file 4: **Table S4.** Normalized expression of miRNA in the midguts of infected and uninfected *Ae. albopictus* at different time points after a DENV-2-infected blood meal (A, infected; B, uninfected). (DOCX 38 kb)

Additional file 5: **Table S5.** All miRNAs with significant differences between the midguts of infected and uninfected *Ae. albopictus* at different time points after a DENV-2-infected blood meal. (DOCX 22 kb)

## Abbreviations

miR-375: microRNA-375
miR-970: microRNA-970
miR-980: microRNA-980
miR-989: microRNA-989
C6/36: *Aedes albopictus* clone
DENV: dengue virus
DENV-2: dengue virus 2
DF: dengue fever
sRNA: small RNA
miR-1420b-5p: microRNA-1420b-5p
miR-1767: microRNA-1767
miR-193-5p: microRNA-193-5p
miR-1951: microRNA-1951
miR-19c: microRNA-19c
miR-276-3p: microRNA-276-3p
miR-2941: microRNA-2941
miR-3811e-5p: microRNA-3811e-5p
miR-4110-5p: microRNA-4110-5p
miR-424-3p: microRNA-424-3p
miR-5706: microRNA-5706
miR-6134: microRNA-6134
miR-622: microRNA-622
miR-976-5p: microRNA-976-5p
miR-989: microRNA-989
miR-1273f: microRNA-1273f
miR-4448: microRNA-4448
PCR: polymerase chain reaction
RT: reverse transcription
