# Supplementary material for: Screening for differentially expressed miRNAs in Aedes albopictus (Diptera: Culicidae) exposed to DENV-2 and their effect on replication of DENV-2 in C6/36 cells
Source: Parasit Vectors. 2019 Jan 18;12:44. doi: 10.1186/s13071-018-3261-2 (PMC6339288; doi:10.1186/s13071-018-3261-2)
Supplement: Supplementary file 1 — Table S1. Basic biological information analysis on sRNAs in the midguts from infected and uninfected Ae. albopictus after a DENV-2-infected blood meal. (DOCX 18 kb) [file 13071_2018_3261_MOESM1_ESM.docx]

**Additional file 1: Table S1.** Basic biological information analysis on sRNAs in the midguts from infected and un-infected *Ae. albopictus* post DENV-2 blood meal

| Type | 5A | | 5B | | 7A | | 7B | | 10A | | 10B | |
| --- | --- | --- | --- | --- | --- | --- | --- | --- | --- | --- | --- | --- |
|  | Count | Percent | Count | Percent | Count | Percent | Count | Percent | Count | Percent | Count | Percent |
| total_reads | 12796601 |  | 19748418 |  | 14676956 |  | 13369774 |  | 15060247 |  | 17579792 |  |
| high_quality | 12762619 | 100% | 19724994 | 100% | 14644504 | 100% | 13337935 | 100% | 15029036 | 100% | 17540659 | 100% |
| 3'adapter_null | 7477 | 0.06% | 3660 | 0.02% | 1809 | 0.01% | 2284 | 0.02% | 999 | 0.01% | 2541 | 0.01% |
| insert_null | 3635 | 0.03% | 4402 | 0.02% | 4555 | 0.03% | 6162 | 0.05% | 3512 | 0.02% | 5524 | 0.03% |
| 5'* | 38281 | 0.30% | 43049 | 0.22% | 93508 | 0.64% | 60487 | 0.45% | 47893 | 0.32% | 34480 | 0.20% |
| smaller_than_18 nt | 13208 | 0.10% | 19961 | 0.10% | 35742 | 0.24% | 28186 | 0.21% | 72545 | 0.48% | 24627 | 0.14% |
| polyA | 14681 | 0.12% | 17996 | 0.09% | 13740 | 0.09% | 29911 | 0.22% | 6336 | 0.04% | 28465 | 0.16% |
| clean_reads | 12685337 | 99.39% | 19635926 | 99.55% | 14495150 | 98.98% | 13210905 | 99.05% | 14897751 | 99.13% | 17445022 | 99.45% |
