# Supplementary material for: Screening for differentially expressed miRNAs in Aedes albopictus (Diptera: Culicidae) exposed to DENV-2 and their effect on replication of DENV-2 in C6/36 cells
Source: Parasit Vectors. 2019 Jan 18;12:44. doi: 10.1186/s13071-018-3261-2 (PMC6339288; doi:10.1186/s13071-018-3261-2)
Supplement: Supplementary file 2 — Table S2. Annotations of clean sRNAs from the midguts of infected Ae. albopictus after a DENV-2-infected blood meal. (DOCX 21 kb) [file 13071_2018_3261_MOESM2_ESM.docx]

**Additional file 2: Table S2.** Annotations of clean sRNAs from the midguts of infected *Ae. albopictus* post DENV -2 blood meal

| Type | 5A | | | | 7A | | | | 10A | | | |
| --- | --- | --- | --- | --- | --- | --- | --- | --- | --- | --- | --- | --- |
|  | Unique  sRNAs | Percent (%) | Total  sRNAs | Percent (%) | Unique  sRNAs | Percent (%) | Total  sRNAs | Percent (%) | Unique  sRNAs | Percent (%) | Total  sRNAs | Percent (%) |
| Total | 801130 | 100 | 12685337 | 100 | 1702196 | 100 | 14495150 | 100 | 766988 | 100 | 14897751 | 100 |
| exon_antisense | 337 | 0.04 | 364 | 0 | 1278 | 0.08 | 1636 | 0.01 | 625 | 0.08 | 680 | 0 |
| exon_sense | 3380 | 0.42 | 31579 | 0.25 | 8509 | 0.5 | 75161 | 0.52 | 3597 | 0.47 | 28232 | 0.19 |
| intron_antisense | 424 | 0.05 | 1189 | 0.01 | 1498 | 0.09 | 3113 | 0.02 | 523 | 0.07 | 1568 | 0.01 |
| intron_sense | 2827 | 0.35 | 39843 | 0.31 | 5764 | 0.34 | 33383 | 0.23 | 3265 | 0.43 | 30447 | 0.2 |
| miRNA | 28576 | 3.57 | 1541279 | 12.15 | 32780 | 1.93 | 3729098 | 25.73 | 29551 | 3.85 | 1982903 | 13.31 |
| rRNA | 52270 | 6.52 | 672941 | 5.3 | 51976 | 3.05 | 723667 | 4.99 | 59456 | 7.75 | 950013 | 6.38 |
| repeat | 4730 | 0.59 | 11598 | 0.09 | 16923 | 0.99 | 44400 | 0.31 | 5260 | 0.69 | 15275 | 0.1 |
| snRNA | 1787 | 0.22 | 5647 | 0.04 | 1999 | 0.12 | 7902 | 0.05 | 1934 | 0.25 | 6522 | 0.04 |
| snoRNA | 158 | 0.02 | 372 | 0 | 227 | 0.01 | 1008 | 0.01 | 186 | 0.02 | 455 | 0 |
| tRNA | 7864 | 0.98 | 135173 | 1.07 | 10385 | 0.61 | 158231 | 1.09 | 10106 | 1.32 | 187329 | 1.26 |
| unann | 698777 | 87.22 | 10245352 | 80.77 | 1570857 | 92.28 | 9717551 | 67.04 | 652485 | 85.07 | 11694327 | 78.5 |

Table S2 (continue). Annotations of clean sRNAs from the midguts of un-infected *Ae. albopictus* post DENV-2 blood meal

| Type | 5B | | | | 7B | | | | 10B | | | |
| --- | --- | --- | --- | --- | --- | --- | --- | --- | --- | --- | --- | --- |
|  | Unique  sRNAs | Percent (%) | Total  sRNAs | Percent (%) | Unique  sRNAs | Percent (%) | Total  sRNAs | Percent (%) | Unique  sRNAs | Percent (%) | Total  sRNAs | Percent (%) |
| Total | 931894 | 100 | 19635926 | 100 | 1408160 | 100 | 13210905 | 100 | 992226 | 100 | 17445022 | 100 |
| exon_antisense | 591 | 0.06 | 658 | 0 | 966 | 0.07 | 1177 | 0.01 | 555 | 0.06 | 664 | 0 |
| exon_sense | 4753 | 0.51 | 53146 | 0.27 | 9347 | 0.66 | 76592 | 0.58 | 4106 | 0.41 | 54989 | 0.32 |
| intron_antisense | 607 | 0.07 | 1669 | 0.01 | 1207 | 0.09 | 3138 | 0.02 | 470 | 0.05 | 2407 | 0.01 |
| intron_sense | 3395 | 0.36 | 83769 | 0.43 | 5987 | 0.43 | 84071 | 0.64 | 3293 | 0.33 | 116920 | 0.67 |
| miRNA | 31034 | 3.33 | 2649446 | 13.49 | 30175 | 2.14 | 2824958 | 21.38 | 28824 | 2.9 | 1886909 | 10.82 |
| rRNA | 53413 | 5.73 | 927720 | 4.27 | 82411 | 5.85 | 1730160 | 13.1 | 63023 | 6.35 | 925612 | 5.31 |
| repeat | 6484 | 0.7 | 18912 | 0.1 | 13055 | 0.93 | 39299 | 0.3 | 5960 | 0.6 | 19824 | 0.11 |
| snRNA | 1988 | 0.21 | 7233 | 0.04 | 3330 | 0.24 | 15313 | 0.12 | 1935 | 0.2 | 5839 | 0.03 |
| snoRNA | 189 | 0.02 | 459 | 0 | 371 | 0.03 | 1229 | 0.01 | 196 | 0.02 | 351 | 0 |
| tRNA | 8995 | 0.97 | 184029 | 0.94 | 15266 | 1.08 | 359491 | 2.72 | 12909 | 1.3 | 159176 | 0.91 |
| unann | 820445 | 88.04 | 15708885 | 80 | 1246045 | 88.49 | 8075477 | 61.13 | 870955 | 87.78 | 14272331 | 81.81 |
