# Supplementary material for: Screening for differentially expressed miRNAs in Aedes albopictus (Diptera: Culicidae) exposed to DENV-2 and their effect on replication of DENV-2 in C6/36 cells
Source: Parasit Vectors. 2019 Jan 18;12:44. doi: 10.1186/s13071-018-3261-2 (PMC6339288; doi:10.1186/s13071-018-3261-2)
Supplement: Supplementary file 3 — Table S3. Matching of small RNAs on the genome of Ae. aegypti in the midguts of infected and uninfected Ae. albopictus at different time points after a DENV-2-infected blood meal. (DOCX 15 kb) [file 13071_2018_3261_MOESM3_ESM.docx]

**Additional file 3: Table S3.** Matching of small RNAs on the genome of *Ae. aegypti* in the midguts of infected and uninfected *Ae. albopictus* at different timepoints post DENV-2 blood meal

| Group | Item | Unique  sRNAs | Percent  (%) | Total  sRNAs | Percent  (%) |
| --- | --- | --- | --- | --- | --- |
| 5A | Total sRNAs | 801130 | 100 | 12685337 | 100 |
|  | Mapping to genome | 54924 | 6.86 | 1589345 | 12.53 |
| 5B | Total sRNAs | 931894 | 100 | 19635926 | 100 |
|  | Mapping to genome | 60355 | 6.48 | 2467874 | 12.57 |
| 7A | Total sRNAs | 1702196 | 100 | 14495150 | 100 |
|  | Mapping to genome | 82371 | 4.84 | 3528185 | 24.34 |
| 7B | Total sRNAs | 1408160 | 100 | 13210905 | 100 |
|  | Mapping to genome | 92691 | 6.58 | 4199905 | 31.79 |
| 10A | Total sRNAs | 766988 | 100 | 14897751 | 100 |
|  | Mapping to genome | 58174 | 7.58 | 2063454 | 13.85 |
| 10B | Total sRNAs | 992226 | 100 | 17445022 | 100 |
|  | Mapping to genome | 62379 | 6.29 | 2051613 | 11.76 |
