# Supplementary material for: Screening for differentially expressed miRNAs in Aedes albopictus (Diptera: Culicidae) exposed to DENV-2 and their effect on replication of DENV-2 in C6/36 cells
Source: Parasit Vectors. 2019 Jan 18;12:44. doi: 10.1186/s13071-018-3261-2 (PMC6339288; doi:10.1186/s13071-018-3261-2)
Supplement: Supplementary file 4 — Table S4. Normalized expression of miRNA in the midguts of infected and uninfected Ae. albopictus at different time points after a DENV-2-infected blood meal (A, infected; B, uninfected). (DOCX 38 kb) [file 13071_2018_3261_MOESM4_ESM.docx]

**Additional file 4: Table S4.** Normalized expression of miRNA in the midguts of infected and un-infected *Ae. albopictus* at different timepoints post DENV-2 blood meal (A. infected, B. un-infected)

| miR Name | 5B | 5A | 7B | 7A | 10B | 10A |
| --- | --- | --- | --- | --- | --- | --- |
| aal-bantam-3p | 75.5 | 89.9 | 305.9 | 322.1 | 87.6 | 88.2 |
| aal-bantam-5p | 1.0 | 1.6 | 3.2 | 3.0 | 1.0 | 1.5 |
| aal-let-7 | 693.2 | 482.8 | 1661.8 | 2387.1 | 594.2 | 625.8 |
| aal-let-7-3p | 0.2 | 0.4 | 0.4 | 0.4 | 0.1 | 0.0 |
| aal-let-7b-5p | 26.2 | 24.1 | 0.5 | 1.0 | 0.3 | 0.4 |
| aal-let-7f | 6.4 | 18.8 | 1.0 | 5.5 | 1.9 | 0.9 |
| aal-miR-1 | 11.9 | 13.0 | 16.5 | 30.6 | 4.6 | 12.6 |
| aal-miR-1-3p | 8314.5 | 5764.0 | 11855.3 | 17921.8 | 3853.2 | 6240.9 |
| aal-miR-1000-5p | 0.5 | 0.7 | 3.1 | 2.6 | 0.3 | 1.1 |
| aal-miR-100-3p | 51.8 | 45.2 | 119.3 | 156.0 | 64.8 | 64.1 |
| aal-miR-100-5p | 45.5 | 46.6 | 112.9 | 114.3 | 23.0 | 47.7 |
| aal-miR-103-3p | 0.5 | 4.9 | 0.0 | 0.7 | 0.0 | 0.1 |
| aal-miR-10-3p | 3.2 | 2.4 | 29.2 | 10.3 | 3.7 | 4.9 |
| aal-miR-10-5p | 0.4 | 0.6 | 2.6 | 1.2 | 0.9 | 0.8 |
| aal-miR-11-3p | 26.2 | 21.7 | 57.5 | 67.8 | 11.9 | 24.4 |
| aal-miR-11-5p | 9.0 | 11.5 | 18.2 | 17.5 | 14.4 | 9.1 |
| aal-miR-1174 | 53.8 | 75.0 | 476.8 | 291.8 | 122.3 | 201.9 |
| aal-miR-1174-3p | 0.3 | 0.2 | 0.3 | 0.2 | 0.2 | 0.2 |
| aal-miR-1175 | 59.8 | 87.4 | 330.2 | 310.4 | 89.0 | 149.1 |
| aal-miR-1175-5p | 887.4 | 926.2 | 1889.7 | 1407.9 | 950.1 | 969.3 |
| aal-miR-122-5p | 0.3 | 3.8 | 0.7 | 0.6 | 1.3 | 0.6 |
| aal-miR-12-3p | 0.2 | 0.4 | 0.6 | 0.8 | 0.2 | 0.3 |
| aal-miR-12-5p | 76.0 | 95.0 | 261.2 | 325.1 | 77.3 | 79.0 |
| aal-miR-124 | 0.0 | 0.0 | 2.3 | 0.3 | 0.3 | 0.3 |
| aal-miR-125-3p | 13.9 | 13.3 | 25.4 | 30.6 | 12.0 | 11.7 |
| aal-miR-125-5p | 19.6 | 27.8 | 61.5 | 64.4 | 14.1 | 27.3 |
| aal-miR-1260 | 3.8 | 8.5 | 35.1 | 11.2 | 3.9 | 29.3 |
| aal-miR-1273f | 37.8 | 37.7 | 137.4 | 35.6 | 52.0 | 76.5 |
| aal-miR-133b | 0.3 | 0.3 | 0.6 | 0.8 | 0.5 | 0.7 |
| aal-miR-13-3p | 20.3 | 20.4 | 87.0 | 73.2 | 28.7 | 23.7 |
| aal-miR-13-5p | 9.1 | 8.8 | 24.8 | 27.5 | 8.2 | 10.3 |
| aal-miR-1357 | 38.1 | 40.9 | 97.1 | 43.0 | 23.0 | 31.6 |
| aal-miR-137-1-5p | 0.6 | 0.1 | 1.2 | 1.5 | 0.9 | 0.4 |
| aal-miR-137-3p | 3.1 | 2.9 | 9.2 | 9.3 | 2.6 | 3.2 |
| aal-miR-14 | 38.2 | 55.7 | 119.8 | 100.8 | 37.0 | 81.1 |
| aal-miR-1420b-5p | 192.3 | 279.3 | 0.4 | 84.6 | 116.4 | 258.0 |
| aal-miR-1421al-5p | 222.1 | 202.4 | 554.8 | 649.9 | 125.9 | 154.5 |
| aal-miR-15-3p | 149.5 | 159.2 | 371.1 | 495.5 | 51.2 | 125.8 |
| aal-miR-15b | 0.4 | 0.2 | 1.9 | 0.6 | 0.4 | 0.8 |
| aal-miR-1587 | 456.5 | 438.7 | 1293.6 | 1167.1 | 410.0 | 411.9 |
| aal-miR-1614-3p | 133.8 | 124.9 | 396.3 | 448.1 | 39.1 | 109.1 |
| aal-miR-1767 | 4808.2 | 3830.3 | 4.2 | 1553.6 | 2024.3 | 4811.7 |
| aal-miR-184-3p | 12615.8 | 11758.5 | 31463.2 | 35918.2 | 13811.0 | 13659.2 |
| aal-miR-184-5p | 0.2 | 0.7 | 1.4 | 1.2 | 0.1 | 0.8 |
| aal-miR-1889-3p | 0.8 | 1.7 | 3.0 | 2.9 | 0.6 | 1.4 |
| aal-miR-1889-5p | 2.0 | 1.5 | 7.0 | 9.2 | 2.2 | 1.5 |
| aal-miR-1890 | 0.3 | 0.6 | 1.3 | 0.9 | 0.3 | 0.4 |
| aal-miR-1891 | 0.1 | 0.5 | 1.1 | 1.2 | 0.1 | 0.0 |
| aal-miR-190-3p | 0.6 | 0.5 | 1.2 | 2.0 | 0.9 | 0.2 |
| aal-miR-190-5p | 36.8 | 35.8 | 101.7 | 95.0 | 34.4 | 26.1 |
| aal-miR-193 | 0.2 | 0.9 | 0.0 | 0.0 | 0.0 | 0.0 |
| aal-miR-193-5p | 1124.8 | 1171.7 | 1.5 | 266.4 | 1567.9 | 1480.1 |
| aal-miR-1951 | 1260.4 | 1352.9 | 1.0 | 363.1 | 1565.8 | 1602.7 |
| aal-miR-1957a | 12.7 | 22.6 | 34.4 | 17.0 | 10.0 | 18.3 |
| aal-miR-19c | 637.0 | 637.0 | 0.6 | 152.6 | 355.8 | 724.1 |
| aal-miR-210-3p | 0.1 | 0.1 | 0.5 | 0.1 | 0.1 | 0.1 |
| aal-miR-2356 | 227.3 | 199.1 | 674.1 | 798.5 | 161.2 | 178.6 |
| aal-miR-241-5p | 434.3 | 436.5 | 1100.2 | 508.9 | 371.5 | 594.9 |
| aal-miR-2423 | 24.6 | 18.4 | 65.0 | 73.7 | 13.0 | 16.2 |
| aal-miR-2449 | 611.8 | 524.5 | 1659.2 | 1888.6 | 426.5 | 549.3 |
| aal-miR-252-5p | 0.4 | 0.9 | 2.4 | 1.6 | 0.9 | 1.0 |
| aal-miR-252b | 0.5 | 0.6 | 1.4 | 1.7 | 0.3 | 0.7 |
| aal-miR-25-3p | 0.0 | 0.7 | 0.1 | 0.0 | 0.0 | 0.1 |
| aal-miR-25-5p | 0.1 | 0.0 | 0.0 | 0.1 | 0.0 | 0.0 |
| aal-miR-263a-3p | 0.7 | 0.9 | 0.5 | 0.7 | 0.6 | 0.5 |
| aal-miR-263a-5p | 4.6 | 4.3 | 10.8 | 17.1 | 4.6 | 3.7 |
| aal-miR-275-3p | 1613.7 | 1721.5 | 2249.9 | 2548.0 | 817.4 | 876.3 |
| aal-miR-275-5p | 2.4 | 2.8 | 10.7 | 10.3 | 3.2 | 1.6 |
| aal-miR-276-3p | 120.2 | 168.5 | 563.2 | 401.4 | 102.2 | 271.7 |
| aal-miR-276-5p | 90.9 | 107.8 | 234.7 | 267.5 | 54.2 | 65.8 |
| aal-miR-2765 | 2.1 | 2.4 | 11.4 | 12.2 | 5.9 | 9.0 |
| aal-miR-277-3p | 11.3 | 13.6 | 42.5 | 36.2 | 12.3 | 14.7 |
| aal-miR-277-5p | 0.4 | 0.2 | 1.5 | 2.1 | 0.1 | 0.3 |
| aal-miR-2779 | 26.7 | 46.4 | 104.0 | 54.3 | 36.1 | 37.0 |
| aal-miR-278-3p | 0.6 | 0.3 | 1.4 | 1.7 | 0.9 | 0.7 |
| aal-miR-278-5p | 1.2 | 0.7 | 3.6 | 1.2 | 2.2 | 1.8 |
| aal-miR-279 | 0.0 | 0.1 | 0.2 | 0.1 | 0.4 | 0.3 |
| aal-miR-279-3p | 12.5 | 34.4 | 66.9 | 94.6 | 12.3 | 23.8 |
| aal-miR-2796-3p | 358.1 | 405.4 | 842.2 | 947.5 | 323.0 | 343.9 |
| aal-miR-2796-5p | 0.2 | 0.2 | 0.5 | 0.1 | 0.1 | 0.1 |
| aal-miR-281-2-5p | 4029.7 | 3982.9 | 11413.1 | 13039.1 | 3968.2 | 4287.3 |
| aal-miR-281-3p | 41.1 | 34.7 | 98.4 | 127.4 | 39.5 | 34.2 |
| aal-miR-283 | 14.7 | 15.0 | 46.9 | 55.5 | 9.9 | 18.1 |
| aal-miR-283-5p | 14.5 | 14.8 | 46.6 | 55.4 | 10.0 | 17.9 |
| aal-miR-285 | 0.1 | 0.2 | 0.2 | 0.4 | 0.1 | 0.0 |
| aal-miR-2940-3p | 198.1 | 211.8 | 391.3 | 521.3 | 200.5 | 201.9 |
| aal-miR-2940-5p | 5745.0 | 5794.6 | 4905.3 | 6784.1 | 4732.8 | 4540.6 |
| aal-miR-2941 | 2.4 | 10.1 | 37.7 | 197.2 | 2.6 | 2.4 |
| aal-miR-2942 | 7.1 | 7.5 | 13.2 | 13.1 | 6.2 | 5.2 |
| aal-miR-2943 | 2.1 | 1.3 | 8.6 | 5.9 | 1.7 | 1.1 |
| aal-miR-2944 | 0.0 | 0.0 | 0.0 | 0.5 | 3.4 | 0.0 |
| aal-miR-2944-5p | 0.7 | 1.3 | 9.8 | 32.4 | 0.3 | 0.5 |
| aal-miR-2945-3p | 428.4 | 464.7 | 1076.1 | 1570.2 | 407.9 | 333.5 |
| aal-miR-2945-5p | 0.4 | 0.2 | 0.8 | 0.4 | 0.8 | 0.5 |
| aal-miR-2946 | 0.4 | 0.3 | 1.6 | 13.2 | 0.5 | 0.1 |
| aal-miR-2951-5p | 315.1 | 334.1 | 827.6 | 374.0 | 180.5 | 443.8 |
| aal-miR-2-5p | 16.6 | 8.0 | 21.3 | 15.9 | 16.1 | 14.6 |
| aal-miR-2a-3p | 25.6 | 29.7 | 76.8 | 82.2 | 18.2 | 42.2 |
| aal-miR-2a-5p | 9.2 | 13.0 | 20.5 | 24.6 | 12.0 | 14.1 |
| aal-miR-2b | 48.4 | 71.0 | 163.0 | 147.9 | 52.3 | 85.2 |
| aal-miR-2c | 24.8 | 28.6 | 74.7 | 79.8 | 17.8 | 41.3 |
| aal-miR-305-3p | 16.7 | 14.3 | 73.0 | 83.1 | 17.6 | 14.8 |
| aal-miR-305-5p | 12.3 | 11.6 | 22.0 | 28.5 | 6.0 | 4.8 |
| aal-miR-306-3p | 0.2 | 0.3 | 0.3 | 0.3 | 0.3 | 0.4 |
| aal-miR-306-5p | 1.6 | 2.8 | 10.7 | 10.0 | 1.8 | 3.8 |
| aal-miR-308 | 0.5 | 0.6 | 4.3 | 3.4 | 3.7 | 0.8 |
| aal-miR-308-5p | 590.3 | 655.4 | 1202.7 | 1334.4 | 746.2 | 712.7 |
| aal-miR-3100-5p | 1826.9 | 1493.9 | 4692.3 | 5281.6 | 1256.9 | 1301.5 |
| aal-miR-31-3p | 2.1 | 2.3 | 5.1 | 7.0 | 0.8 | 3.4 |
| aal-miR-31-5p | 215.9 | 238.8 | 433.7 | 465.2 | 200.7 | 227.8 |
| aal-miR-316 | 16.8 | 25.9 | 72.4 | 38.0 | 30.3 | 45.9 |
| aal-miR-317 | 2696.1 | 3333.3 | 6340.6 | 6180.9 | 908.7 | 3196.5 |
| aal-miR-317-5p | 2.0 | 3.2 | 4.9 | 5.0 | 2.5 | 3.1 |
| aal-miR-33-5p | 65.1 | 76.5 | 159.2 | 241.9 | 66.8 | 29.5 |
| aal-miR-34-3p | 2.1 | 4.7 | 16.9 | 8.1 | 4.2 | 12.1 |
| aal-miR-34-5p | 5860.2 | 5439.0 | 18287.9 | 19916.7 | 7354.9 | 8090.1 |
| aal-miR-375 | 0.7 | 2.3 | 0.0 | 0.0 | 0.3 | 0.0 |
| aal-miR-3809-3p | 0.6 | 0.5 | 2.0 | 1.6 | 1.8 | 1.9 |
| aal-miR-3809-5p | 394.3 | 378.4 | 816.4 | 365.1 | 354.4 | 486.3 |
| aal-miR-3811e-5p | 846.2 | 754.8 | 0.8 | 181.3 | 1162.4 | 1098.0 |
| aal-miR-3870-5p | 324.9 | 275.8 | 887.2 | 1015.9 | 35.3 | 234.3 |
| aal-miR-3888-5p | 84.3 | 131.6 | 302.3 | 68.9 | 118.1 | 97.7 |
| aal-miR-4110-5p | 160.6 | 146.2 | 0.2 | 44.9 | 260.8 | 56.1 |
| aal-miR-4175-3p | 6656.9 | 7506.9 | 17592.3 | 6626.1 | 5275.9 | 8820.3 |
| aal-miR-424-3p | 869.2 | 911.5 | 1.0 | 220.8 | 957.5 | 246.7 |
| aal-miR-4443 | 29.8 | 27.1 | 49.3 | 63.0 | 43.5 | 45.0 |
| aal-miR-4448 | 114.9 | 109.8 | 1009.6 | 132.3 | 118.1 | 101.5 |
| aal-miR-4728-5p | 791.3 | 645.6 | 1.5 | 210.7 | 443.8 | 1089.2 |
| aal-miR-493-3p | 1054.5 | 920.6 | 2775.9 | 2968.4 | 543.7 | 576.3 |
| aal-miR-493-5p | 0.9 | 1.7 | 5.8 | 1.4 | 1.8 | 2.6 |
| aal-miR-5706 | 3953.1 | 3705.0 | 9369.1 | 10276.8 | 273.6 | 2434.3 |
| aal-miR-6086 | 234.5 | 199.2 | 589.2 | 744.2 | 184.6 | 188.0 |
| aal-miR-6134 | 856.1 | 535.5 | 1.3 | 258.1 | 749.0 | 281.9 |
| aal-miR-622 | 16264.1 | 10635.2 | 365.0 | 6475.0 | 18758.4 | 15834.6 |
| aal-miR-6666-3p | 8.5 | 1.8 | 69.7 | 18.3 | 43.0 | 46.2 |
| aal-miR-6668-3p | 608.2 | 493.4 | 1349.5 | 1679.0 | 214.5 | 403.6 |
| aal-miR-7 | 2.9 | 2.4 | 8.5 | 8.1 | 2.8 | 2.3 |
| aal-miR-71-3p | 0.8 | 2.5 | 8.3 | 4.9 | 0.9 | 8.2 |
| aal-miR-71-5p | 224.0 | 182.4 | 656.0 | 768.1 | 172.8 | 300.6 |
| aal-miR-79 | 46.0 | 48.2 | 120.7 | 114.7 | 20.9 | 35.2 |
| aal-miR-79-3p | 45.5 | 47.1 | 119.1 | 112.7 | 20.6 | 34.8 |
| aal-miR-8-3p | 519.6 | 545.7 | 1429.3 | 1697.1 | 443.9 | 483.5 |
| aal-miR-8-5p | 178.6 | 139.5 | 351.5 | 511.0 | 135.4 | 115.7 |
| aal-miR-9 | 11.5 | 6.5 | 27.7 | 35.2 | 6.8 | 9.5 |
| aal-miR-927-3p | 1.9 | 3.4 | 6.6 | 2.8 | 2.4 | 3.0 |
| aal-miR-927-5p | 0.2 | 0.2 | 0.4 | 0.4 | 0.1 | 0.1 |
| aal-miR-92a | 15.7 | 16.6 | 44.1 | 37.3 | 19.9 | 19.1 |
| aal-miR-92a-5p | 14.7 | 16.1 | 34.9 | 26.4 | 16.5 | 15.6 |
| aal-miR-92b | 8.7 | 8.4 | 21.5 | 22.8 | 9.2 | 11.4 |
| aal-miR-932-3p | 0.8 | 0.8 | 1.7 | 1.6 | 0.3 | 0.4 |
| aal-miR-956-3p | 20364.1 | 17277.0 | 64589.2 | 73482.1 | 11388.5 | 17946.3 |
| aal-miR-956-5p | 10.0 | 8.5 | 12.6 | 16.7 | 8.7 | 3.6 |
| aal-miR-965 | 1.1 | 1.4 | 6.1 | 4.2 | 2.9 | 3.7 |
| aal-miR-970-3p | 368.6 | 326.9 | 1141.6 | 1078.3 | 618.3 | 661.8 |
| aal-miR-976-5p | 690.1 | 868.7 | 1.4 | 369.0 | 1401.3 | 1467.5 |
| aal-miR-980-3p | 0.1 | 0.0 | 0.3 | 0.3 | 0.1 | 0.0 |
| aal-miR-980-5p | 0.5 | 0.4 | 2.5 | 1.7 | 1.7 | 1.4 |
| aal-miR-988-3p | 3.5 | 4.3 | 17.0 | 11.2 | 1.9 | 8.3 |
| aal-miR-989 | 8.5 | 5.7 | 102.6 | 904.8 | 3.7 | 6.1 |
| aal-miR-993 | 0.1 | 0.1 | 0.8 | 0.4 | 0.8 | 0.1 |
| aal-miR-993-3p | 0.0 | 0.2 | 1.0 | 0.8 | 0.5 | 0.3 |
| aal-miR-996 | 77.8 | 96.1 | 241.5 | 285.1 | 32.3 | 74.0 |
| aal-miR-998-3p | 22.2 | 27.4 | 89.5 | 93.2 | 27.8 | 46.6 |
| aal-miR-998-5p | 59.0 | 73.5 | 139.7 | 152.8 | 12.6 | 49.7 |
| aal-miR-999-3p | 6.4 | 5.5 | 15.7 | 16.0 | 6.3 | 4.0 |
| aal-miR-9a-3p | 1.4 | 1.4 | 7.0 | 3.3 | 1.4 | 2.1 |
| aal-miR-9a-5p | 11.4 | 6.4 | 26.4 | 32.8 | 6.4 | 9.0 |
| aal-miR-9b-3p | 44.6 | 46.2 | 116.4 | 110.2 | 20.7 | 34.0 |
| aal-miR-9b-5p | 4.0 | 3.5 | 6.9 | 9.6 | 2.1 | 2.8 |
| aal-miR-9c-3p | 10.0 | 14.0 | 30.7 | 30.3 | 6.9 | 13.4 |
| aal-miR-9c-5p | 29.6 | 32.5 | 71.8 | 87.8 | 18.1 | 22.3 |
| aal-miR-9d-3p | 44.5 | 46.4 | 116.5 | 110.7 | 20.7 | 34.3 |
| aal-miR-iab-4-3p | 0.4 | 0.6 | 1.2 | 1.9 | 0.6 | 0.3 |
| aal-miR-iab-4-5p | 2.5 | 4.0 | 8.3 | 8.1 | 2.4 | 2.9 |
