# Supplementary material for: Screening for differentially expressed miRNAs in Aedes albopictus (Diptera: Culicidae) exposed to DENV-2 and their effect on replication of DENV-2 in C6/36 cells
Source: Parasit Vectors. 2019 Jan 18;12:44. doi: 10.1186/s13071-018-3261-2 (PMC6339288; doi:10.1186/s13071-018-3261-2)
Supplement: Supplementary file 5 — Table S5. All miRNAs with significant differences between the midguts of infected and uninfected Ae. albopictus at different time points after a DENV-2-infected blood meal. (DOCX 22 kb) [file 13071_2018_3261_MOESM5_ESM.docx]

**Additional file 5: Table S5.** All miRNAs with significant differences between the midguts of infected and un-infected *Ae. albopictus* at different timepoints post DENV-2 blood meal

| miR name | 5dpe | | | 7dpe | | | 10dpe | | |
| --- | --- | --- | --- | --- | --- | --- | --- | --- | --- |
|  | 5B | 5A | FC | 7B | 7A | FC | 10B | 10A | FC |
| let-7f | 6.4 | 18.8 | 1.5 | 1.0 | 5.5 | 2.5 | - | - | - |
| miR-1 | - | - | - | - | - | - | 4.6 | 12.6 | 1.5 |
| miR-100 | - | - | - | - | - | - | 23.4 | 48.6 | 1.1 |
| miR-10-3p | - | - | - | 29.2 | 10.3 | -1.5 | - | - | - |
| miR-11 | - | - | - | - | - | - | 11.8 | 24.7 | 1.1 |
| miR-12 | - | - | - | - | - | - | 8.9 | 19.9 | 1.2 |
| miR-1260 | 3.8 | 8.5 | 1.2 | 35.1 | 11.2 | -1.7 | 3.9 | 29.3 | 2.9 |
| miR-1273f | - | - | - | 137.4 | 35.6 | -1.9 | - | - | - |
| miR-14 | - | - | - | - | - | - | 37.0 | 81.1 | 1.1 |
| miR-1420b-5p | - | - | - | 0.4 | 84.6 | 7.8 | 116.4 | 258.0 | 1.1 |
| miR-15-3p | - | - | - | - | - | - | 51.2 | 125.8 | 1.3 |
| miR-1767 | - | - | - | 4.2 | 1553 | 8.5 | 2024 | 4811 | 1.2 |
| miR-193-5p | - | - | - | 1.5 | 266.4 | 7.5 | - | - | - |
| miR-1951 | - | - | - | 1.0 | 363.1 | 8.5 | - | - | - |
| miR-1957b | - | - | - | 73.0 | 28.1 | -1.4 | - | - | - |
| miR-19c | - | - | - | 0.6 | 152.6 | 8.0 | 355.8 | 724.1 | 1.0 |
| miR-241-5p | - | - | - | 1100 | 508.1 | -1.1 | - | - | - |
| miR-2-5p | 16.6 | 8.0 | -1.1 | - | - | - | - | - | - |
| miR-276-3p | 120.2 | 337.1 | 1.5 | 153.3 | 1711 | 2.9 | 102.2 | 271.7 | 1.4 |
| miR-2941 | 2.4 | 10.1 | 2.0 | 37.7 | 197.2 | 2.4 | - | - | - |
| miR-2944b-5p | - | - | - | 9.8 | 32.4 | 1.7 | - | - | - |
| miR-2946 | - | - | - | 1.6 | 13.2 | 3.1 | - | - | - |
| miR-2951-5p | - | - | - | 827.6 | 374.0 | -1.1 | 180.5 | 443.8 | 1.3 |
| miR-2a-3p | - | - | - | - | - | - | 182. | 42.2 | 1.2 |
| miR-2c | - | - | - | - | - | - | 17.8 | 41.3 | 1.2 |
| miR-317 | - | - | - | - | - | - | 908.7 | 3197 | 1.8 |
| miR-33 | - | - | - | - | - | - | 68.3 | 29.7 | -1.2 |
| miR-34-3p | 2.1 | 4.7 | 1.2 | 16.9 | 8.1 | -1.1 | 4.2 | 12.1 | 1.5 |
| miR-3809-5p | - | - | - | 816.4 | 365.1 | -1.2 | - | - | - |
| miR-3811e-5p | - | - | - | 0.8 | 181.3 | 7.8 | - | - | - |
| -miR-3870-5p | - | - | - | - | - | - | 35.3 | 234.3 | 2.7 |
| miR-4110-5p | - | - | - | 0.2 | 44.9 | 7.6 | 56.1 | 260.8 | 2.2 |
| miR-4175-3p | - | - | - | 17592 | 6626 | -1.4 | - | - | - |
| miR-424-3p | - | - | - | 1.0 | 220.8 | 7.8 | 246.7 | 957.5 | 2.0 |
| miR-4448 | 206.9 | 109.8 | -1.0 | 1009 | 132.3 | -2.9 | - | - | - |
| miR-4728-5p | - | - | - | 1.5 | 210.7 | 7.1 | 443.8 | 1089 | 1.3 |
| miR-5706 | - | - | - | - | - | - | 273.6 | 2434 | 3.2 |
| miR-6134 | - | - | - | 1.3 | 258.1 | 7.6 | 281.9 | 749.0 | 1.4 |
| miR-622 | 4337 | 10635 | 1.3 | 365 | 6427 | 4.1 | - | - | - |
| miR-6666-3p | 8.5 | 1.8 | -2.2 | 69.7 | 18.3 | -1.9 | - | - | - |
| miR-976-5p | - | - | - | 1.4 | 369 | 8.0 | - | - | - |
| miR-989 | - | - | - | 102.6 | 904.8 | 3.1 | - | - | - |
| miR-996 | - | - | - | - | - | - | 32.3 | 74.0 | 1.2 |
| miR-998-5p | - | - | - | - | - | - | 12.6 | 49.7 | 2.0 |

FC: fold-change
